# Trial Protocol: Reaccumulation rate of pleural effusions after therapeutic aspiration: An observational cohort study to determine baseline factors associated with rate of pleural fluid reaccumulation following therapeutic aspiration in patients with malignant pleural effusion attending a pleural clinic (REPEAT)

**DOI:** 10.3310/nihropenres.13282.1

**Published:** 2023-01-23

**Authors:** Eleanor K. Mishra, Allan Clark, Magda Laskawiec-Szkonter, Nicholas A. Maskell, Najib M. Rahman

**Affiliations:** 1 University of East Anglia, Norwich, NR4 7TL, UK; 2 Norfolk and Norwich University Hospitals NHS, Norwich, UK; 3 Oxford Respiratory Trials Unit, University of Oxford, Oxford, OX3 7LE, UK; 4 University of Bristol, Bristol, BS8 1TH, UK

**Keywords:** Pleural effusion, cancer, therapeutic aspiration, breathlessness

## Abstract

**Background:**

Malignant pleural effusion (MPE) is the build-up of pleural fluid in the space between the lung and chest wall due to advanced cancer. It is treated initially by large volume drainage (therapeutic aspiration). If the fluid reaccumulates, a definitive procedure is performed. There is wide variation in rate of reaccumulation. Patients with rapid reaccumulation often attend hospital as an emergency. Conversely, patients with slow reaccumulation do not need a definitive procedure and may experience cancelled or unnecessary procedures. This study aims to create and validate a multivariable prediction model to predict how quickly pleural fluid will reaccumulate in patients with MPE following therapeutic aspiration.

**Research question:**

Can we predict how quickly pleural fluid will reaccumulate in patients with MPEs?

**Methods:**

A total of 200 patients with known or suspected MPE attending for therapeutic aspiration will be recruited from 5–10 UK hospitals over 20 months. Patients will be enrolled prior to undergoing aspiration. Following this, they will undergo chest X-ray, which will be repeated one week later (treatment as usual). Rate of reaccumulation will be calculated based on change of size of the effusion seen on X-ray. Data will be collected on common clinical biomarkers *e.g.*, size of effusion on pre-aspiration chest X-ray, volume of fluid drained. This data will be analysed to create a clinical score.

A further validation cohort of 40 patients will be enrolled in parallel with creation of the score.

**Anticipated impact:**

The ability to predict rate of reaccumulation of MPE will enable patients and clinicians to make better informed treatment decisions. For patients with predicted rapid reaccumulation, a definitive procedure could be offered as first-line treatment, rather than a therapeutic aspiration. This will prevent emergency hospital admissions and decrease number of procedures. By contrast, patients whose effusions will recur slowly may avoid an unnecessary procedure.

## Plain English summary

People with incurable cancer commonly feel breathless due to buildup of fluid around the lung. We treat this by draining fluid off, but it often comes back. When this happens, we offer the patient a permanent implanted drain, so they can drain the fluid off at home. However, sometimes the fluid builds up very quickly. The patient becomes very breathless and needs an emergency hospital admission. In other people, the fluid builds up slowly and they may never need another drain.

The aim of this study is to improve treatment by finding a way to predict how quickly fluid will come back. It will be run in five hospitals across England and involve 240 patients over three years. When patients first come to have fluid drained, we will record information about them and their disease. We will measure how quickly the fluid comes back and record hospital admissions and need for fluid drainage over the following three months. We will use this information to create a clinical score. We will then test this score in a second group of patients to make sure it works.

The idea for this study came from one of our patients who had just had fluid drained and asked, ‘when will it come back?’ We couldn’t answer his question and therefore developed this study. Our patients and the public have been involved in the design of this study and will continue to be involved.

This score will be used to inform patients so they can choose the best treatment for them. If we know the fluid will build up quickly, we can offer patients a permanent drain straight away. If patients know their fluid will build up slowly, they may choose to have the fluid drained when needed without a permanent drain.

## Introduction

### Background and rationale

Patients with cancer develop malignant pleural effusions (MPEs) when fluid collects between the lung and chest wall (the pleural space). MPEs are common, with more than 30,000 new cases per year in the United Kingdom. They are caused by cancers that have spread to the lining of the pleural space and are common in breast and lung cancer and mesothelioma, a type of cancer caused by exposure to asbestos ^1^. MPE may be the first presentation of cancer or develop in a patient with known cancer, but sadly always means the disease is not curable. Patients experience breathlessness, chest discomfort and cough ^2^.

The development of a pleural effusion occurs due to the excess production of pleural fluid. The pleural space is lined by a single layer of pleural mesothelial cells (PMCs). These regulate the production of pleural fluid through the control of substances passing in between mesothelial cells ( *via* tight junctions) and through cells ^3^. Cancer cells in the pleural space stimulate the production of a pleural effusion *via* inflammatory pathways and release of vasoactive mediators, such as vascular endothelial growth factor (VEGF) ^4^. This increases mesothelial permeability *via* loosening of tight junctions between PMCs, allowing the passage of proteins and fluid into the pleural space. VEGF levels are raised in MPEs ^5^. However, the mechanisms are complex and not yet fully understood.

Treatment of MPE aims to effectively relieve symptoms, prevent emergency hospital admission and reduce the number of procedures and cancelled procedures. Patients usually attend a specialist Pleural Clinic as an outpatient for an initial drainage procedure (therapeutic aspiration). This is an effective, simple and quick way of treating symptoms. However, this does not treat the underlying cause of fluid accumulation. Pleural fluid usually comes back, which results in more breathlessness. When this happens, a definitive procedure (chest drain and pleurodesis or indwelling pleural catheter insertion) is performed ^6^.

Our pilot data demonstrates that the fluid comes back at a very variable rate. Some patients produce pleural fluid quickly and develop rapid onset, severe breathlessness, which results in an emergency hospital attendance. By contrast, some effusions develop more slowly, and patients may attend for a planned drainage procedure that has to be cancelled due to lack of fluid or have an unnecessary procedure. Currently, there is no way to predict the rate of fluid recurrence for each individual patient.

In summary, MPEs are a common cause of breathlessness in patients with advanced cancer. There is wide variation in the rate of fluid accumulation. Rapid accumulation can result in an emergency hospital admission whereas slow accumulation can lead to unnecessary or cancelled procedures.

***Why is this research important in terms of improving the health of patients and improving health care services?***

This research is important to improve the care offered to patients with MPEs. As part of the pilot work for this study, we surveyed pleural clinicians involved in deciding when patients undergo a definitive pleural procedure for MPE. Over half (58%) reported that their patients often or sometimes get admitted as an emergency with severe breathlessness caused by recurrent MPE. Over one quarter of cancer patients admitted as an emergency die in hospital ^7^. Patients do not want hospital admissions, find them tiring and experience unnecessary delays ^8, 9^. The results of this research will enable us to identify patients with rapid pleural fluid accumulation so they can undergo an early definitive pleural procedure and prevent emergency hospital admissions.

 This research will also identify patients whose MPE will reaccumulate slowly or not at all. These patients may currently undergo unnecessary definitive pleural procedures or have their procedure cancelled due to lack of pleural fluid. Our survey data demonstrated that 45% of pleural clinicians often or sometimes cancel a pleural procedure due to lack of fluid. This is inconvenient and distressing for the patient.

 Our research will also help clinicians plan pleural services, by identifying which patients require an urgent procedure and reducing unnecessary and cancelled procedures. Our survey revealed that 94% of pleural clinicians thought a score that enabled them to predict how quickly MPEs would recur would help plan their service.

***Review of existing evidence***

There has been little research to date studying predictors of pleural fluid reaccumulation. One previous retrospective cohort study found that the size of effusion on CXR prior to therapeutic aspiration, the volume of pleural fluid drained during therapeutic aspiration, the pleural fluid lactate dehydrogenase (LDH) levels and positive pleural fluid cytology predicted a further pleural drainage procedure being carried out in a single centre with a highly protocol-led pathway ^10^. However, these variables were not predictive in other centres. This paper demonstrated wide variation in time to a further drainage procedure, with 30% of patients requiring a further procedure within 15 days whereas 52% had not had another procedure at 90 days. Boshuizen *et al.*, also found that the volume of pleural fluid drained was predictive of need for a further pleural intervention ^11^.

 Despite the lack of evidence for predictors of pleural fluid recurrence, our survey demonstrated that 71% of clinicians try to predict how quickly pleural fluid will recur. However, there was no consistent method for doing this. Clinicians based this judgement on variables such as the speed of onset of symptoms, initial size of the effusion, tumour type and previous recurrence rate (based on review of previous radiology/time between previous drainages).

***Data from feasibility study***

We successfully recruited 20 patients to a feasibility study at the Norfolk and Norwich Pleural Unit from January to July 2019. We screened 40 patients attending the Pleural Clinic for a therapeutic aspiration of their effusion for eligibility and identified 23 eligible patients. Of these, 20 consented to enrolment. All enrolled patients attended the one-week primary outcome follow-up. The feasibility study included qualitative interviewing of the patients and patients were asked about their experience of the study as part of this interviewing. Our results demonstrated that this study is acceptable to patients and recruitment to target is feasible.

The feasibility study identified that only 15 of these 20 patients subsequently underwent therapeutic aspiration. This was because thoracic ultrasound (US) performed following enrolment demonstrated that there was insufficient fluid for a therapeutic aspiration or that the fluid was not amenable to drainage. Therefore, we altered the inclusion criteria to specify that patients must have an effusion demonstrated on thoracic US or computer tomography (CT) scan prior to enrolment. This change to the inclusion criteria will ensure that all enrolled patients will undergo therapeutic aspiration.

We plan to analyse data only on patients with a final diagnosis of an MPE because effusions from other causes usually resolve with treatment of the underlying cause and recurrence is not a problem. However, when patients attend for therapeutic aspiration, the final diagnosis may not be known. Of the 15 patients who underwent therapeutic aspiration in our feasibility study, 12 of them had a final diagnosis of malignancy. For the full study, we plan to recruit 200 patients in Phase 1 to include at least 150 with a final diagnosis of MPE and full primary outcome data.

### Objectives and outcome measures

The primary objective of this study is **t**o identify candidate biomarkers associated with the rate of pleural fluid accumulation in patients with MPE and to use these biomarkers to develop and validate a clinical score to predict rate of reaccumulation. These biomarkers will be measured at day 0. This will be compared with the rate of pleural fluid accumulation based on increased size of pleural effusion from CXR immediately post-aspiration to day 7 ^12^.

*Complete list of candidate biomarkers to be assessed for the primary objective*

The candidate biomarkers include:

- Patient biomarkers: i) duration of symptoms; type of malignancy; ii) known malignancy or first presentation; iii) severity of pre-procedure breathlessness (measured on visual analogue scale); iv) previous chemotherapy and/or radiotherapy and/or immunotherapy; v) current chemotherapy and/or radiotherapy and/or immunotherapy; vi) current use of diuretics; emergency ( *via* Accident and Emergency (A&E) or acute medical unit) or urgent presentation ( *via* General Practice (GP) or oncology); vii) performance status (assessed using Eastern Co-operative Group scale); and viii) co-morbidities (chronic renal failure, liver disease, cardiac failure).
- Effusion biomarkers: i) size of effusion on pre-procedure CXR (measured as percentage opacification of hemithorax - this is a validated measure of effusion size); ii) size of effusion on pre-procedure US (measured from hemidiaphragm to top of effusion); iii) side of effusion; iv) volume of fluid drained during therapeutic aspiration (ml); v) degree of pleural fluid septations (categorised as none, mild, moderate or severe) on US; vi) significant non-expandable lung on post-procedure CXR; and vii) pleural pressures measured by manometry (substudy in 20 patients only in Norwich).
- Pleural fluid biomarkers: i) total protein; ii) pH; iii) glucose; iv) lactose dehydrogenase (LDH); and v) cytology (categorised as positive *i.e.*, malignant cells present or negative).
- Serum biomarkers: i) haemoglobin; ii) white cell count, including neutrophil and leukocyte subsets; iii) C reactive protein; iv) total protein; v) estimated glomerular filtration rate (eGFR); vi) LDH; and vii) N-terminal pro-brain natriuretic peptide (NT-proBNP).

***Why were these biomarkers chosen?***

Duration of symptoms, underlying diagnosis and pre-procedure breathlessness were common factors identified by our clinician survey as being important in estimating how quickly pleural fluid will recur. Current treatment may reduce how quickly an effusion recurs. The co-morbidities specified are all associated with the production of pleural fluid, which may increase the rate of reaccumulation (despite the main cause being malignancy). Pre-procedure size of effusion was identified on two previous retrospective cohort studies as being associated with the need for a further pleural procedure, and we are measuring this in three ways (on X-ray, US and volume fluid drained). Septations are fibrin or collagen walls that develop within the effusion and impair drainage, and therefore may influence rate of reaccumulation. Non-expandable lung is defined as failure of the lung to re-expand following therapeutic aspiration and will be visible on the post-procedure CXR. It is associated with significant negative pressure within the pleural space, which may drive rapid pleural fluid reaccumulation. pH, LDH and cytology have been identified in previous studies as being associated with the need for a further pleural procedure. Total protein determines whether an effusion is an exudate or transudate and therefore is an important variable determining the mechanism of pleural fluid production. LDH has been identified as a variable associated with poor prognosis in patients with MPE. Haemoglobin, neutrophil: leukocyte ratio and C reactive protein have been shown to be associated with prognosis in patients with MPE. NT-proBNP is a measure of cardiac function and eGFR is a measure of renal function, so these variables may affect pleural fluid reaccumulation. High pleural fluid pressures may be associated with a strong drive to produce pleural fluid and hence rapid reaccumulation. These candidate biomarkers will be assessed to see which are appropriate for further analysis, based on our ability to detect variability within these measurements. If required, principal component analysis will be used to reduce the number of variables to eight for further analysis.

## Protocol

### Study design

This will be an observational cohort study recruiting patients with a pleural effusion attending the pleural clinic at five UK hospitals for a therapeutic aspiration of their effusion for breathlessness relief.

1. **Phase 1:** observational cohort study of 200 patients in five hospitals for initial data to identify baseline characteristics associated with rate of pleural fluid accumulation and develop a predictive score.
2. **Phase 2:** validation observational cohort study of 40 patients in the same five hospitals to validate the score: this will be done in parallel with the analysis of data from phase 1.

If the above two phases are successful, we will proceed to:

3. **Phase 3:** a final observational cohort study of 200 patients in the same five hospitals using the score to demonstrate impact on breathlessness, emergency admissions, number of pleural procedures, quality of life (QoL) and health economic outcomes: this final phase will be funded separately.

This protocol is for the first and second phases of this study.

### Participant identification

***Study participants***

Patients with a pleural effusion attending the pleural clinic at five UK hospitals for a therapeutic aspiration of their effusion with known or suspected MPE.

***Inclusion criteria***

- Participant is willing and able to give informed consent for participation in the study.
- Aged 18 years old or above.
- Diagnosed with pleural effusion on CT or US.
- Patient attending for therapeutic aspiration (large volume drainage) of their pleural effusion: there is no specific minimal pleural fluid volume, but this should be larger than required for diagnosis alone (typically 60 ml is taken for diagnostic purposes).
- Known or suspected malignancy as the underlying cause of the effusion.
- In the Investigator’s opinion, is able and willing to comply with all study requirements.

***Exclusion criteria***

The participant may not enter the study if any of the following apply:

- Patients who are pregnant or lactating.
- Pleural infection or other condition requiring admission and chest drain insertion.
- Known transudative pleural effusion or pleural effusion clinically thought to be primarily due to cardiac, renal or hepatic impairment.

### Protocol procedures

**Figure 1.**
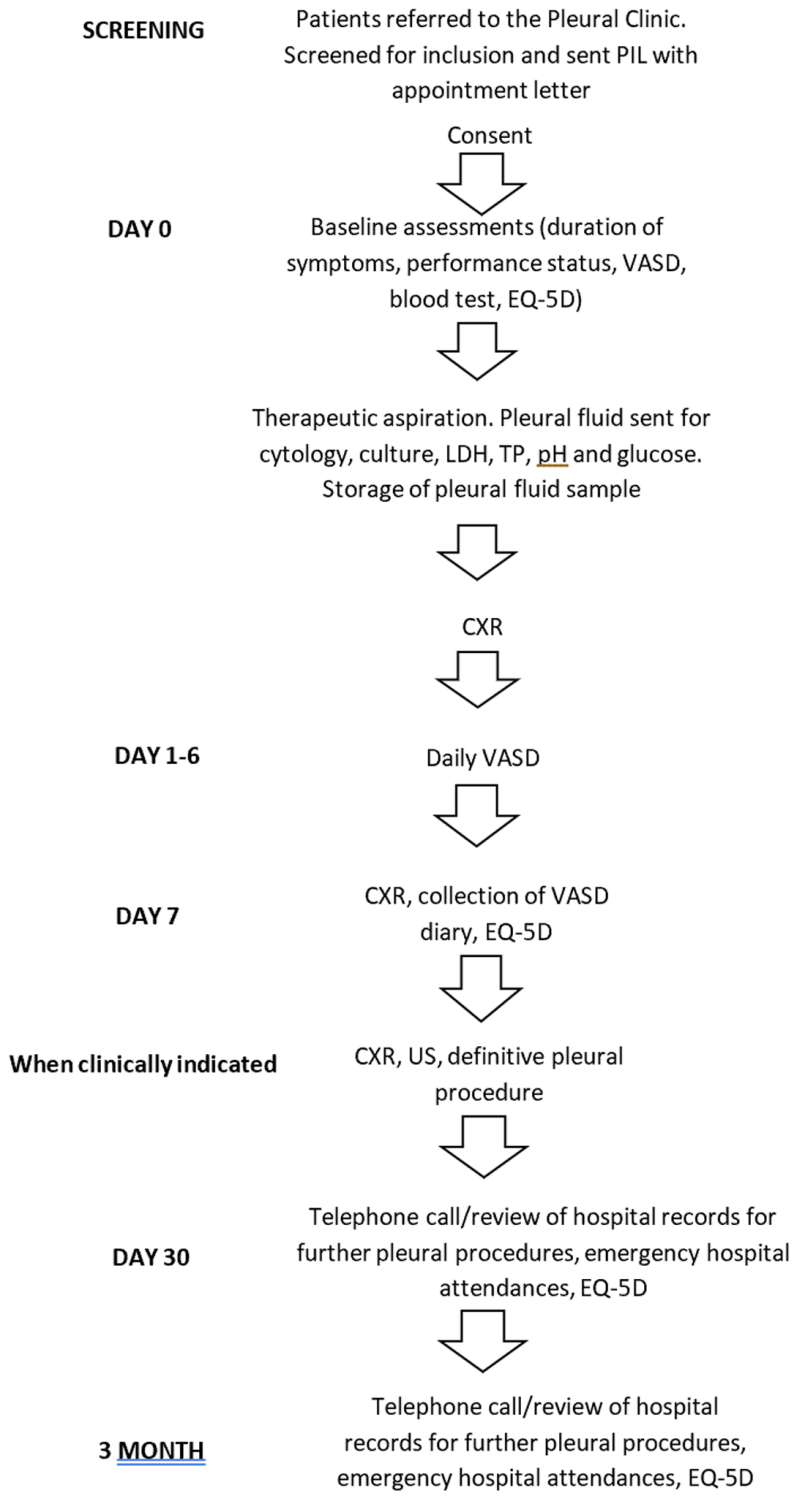
Study flow chart. PIL, patient information leaflet; VASD, visual analogue scale for dyspnoea; EQ-5D, baseline QoL questionnaire; LDH, lactate dehydrogenase; TP, total protein; CXR, chest X-ray; US, ultrasound.

See Figure 1 for study flow chart.

***Recruitment***

All patients attending the Pleural Clinic for a therapeutic aspiration of pleural fluid will be informed of the study when their appointment is booked and will receive a patient information leaflet (PIL) by post with their appointment letter. Those who are interested in participating will be asked to contact the study team to confirm that they wish to take part. If no response is received, patients will be approached prior to their fluid drainage procedure by a member of the study team to confirm if they wish to participate, either by telephone or when they attend pleural clinic.

***Screening and eligibility assessment***

Patients attending the Pleural Clinic for therapeutic aspiration will be screened by confirming they fulfil the inclusion criteria, able to attend the follow-up appointment in one week and are willing and able to give informed consent. Patients will already have had clinical information, blood tests, thoracic US and pre-procedure CXR performed as part of standard clinical care.

***Informed consent***

Patients interested in participating in the study will be contacted by a member of the study team prior to their drainage procedure, either by telephone or at the time of attending pleural clinic. They will be given a copy of the Participant Information Sheet (PIS) and Consent form. This will explain why we are doing the study and what it involves for them. The PIS states that the patient is free to withdraw at any time and this will not affect their medical care or legal rights. The PIS and Consent form used in this study can be found as *Extended data* ^13^.

They will have as much time as they want to think about joining the study and the opportunity to ask the research team any questions. There is no minimum time for this.

The participant must personally sign and date the latest approved version of the Informed Consent form before any study specific procedures are performed. The person who obtained consent must be trained in Good Clinical Practice (GCP) and have been authorised to do so by the Chief/Principal Investigator and must sign and date the informed consent form.

We anticipate that participants may lose capacity during the study due to disease progression. On the consent form, participants will be asked if they are happy for the study team to retain and use the study data and samples collected up to that point.

### Baseline assessments

The following baseline assessments will be made:

- Clinical characteristics: duration of symptoms; Eastern Cooperative Oncology Group performance status (ECOG PS) ^14^; underlying diagnosis, current treatment of effusion *e.g.*, chemotherapy for cancer, presence or absence of contralateral effusion.
- Baseline visual analogue scale for dyspnoea (VASD) (can be found as *Extended data* ^13^).
- Blood samples will be taken and stored (optional long-term storage for future analysis - in selected sites depending on resources available).
- Baseline QoL questionnaire (EQ-5D 5L) ^15^

Patients will then undergo therapeutic aspiration, according to the standard operating procedure. Pleural manometry may be performed during aspiration using bespoke equipment with a manometer integrated in the therapeutic aspiration sheath (supplied by Rocket Medical, optional substudy of 20 patients in Norwich only).

Pleural fluid will be sent to local laboratories for microscopy and culture, cytology, total protein (TP), LDH, pH and glucose. These samples are all part of standard clinical care.

In addition, pleural fluid samples may be stored locally (optional long-term storage for future analysis - in selected sites depending on resources available).

Participants will then have a post-procedure CXR (as standard care) and thoracic US (as a study procedure) to assess size of residual effusion and presence of trapped lung, which will be recorded on the Case Report Forms (CRFs) (for later subgroup analysis) (can be found as *Extended data* ^13^).

Following discharge, they will complete the seven-day VASD questionnaire (7DVQ) over one week on paper: this is a seven-day questionnaire in which patients assess their average level of breathlessness on a visual analogue scale daily.

### Follow-up visits

- **Day seven:** follow-up visit seven days after the therapeutic aspiration (can be done 5–10 days). This is part of standard clinical care for patients to receive results of pleural fluid testing and decide a management plan. Patients standardly have a CXR at this visit and this will be used to reassess size of effusion. At the point, the 7DVQ will be collected and a CRF completed, particularly to assess whether participants have required further pleural fluid drainage or attended the hospital with breathlessness during the seven-day period. During this period the participant is permitted to have any clinically required intervention.
- **Second pleural procedure:** this will be performed if clinically indicated for diagnostic or symptomatic reasons. Indications for symptomatic drainage are as follows: symptomatic benefit from initial pleural fluid drainage; significant breathlessness; and significant pleural effusion (defined as at least 25% of the hemithorax on CXR or estimated volume of pleural fluid of at least 500 ml using US). Participants will undergo a further pleural drainage ( *via* thoracoscopy, chest drain or indwelling pleural catheter insertion according to clinical requirement in their individual care pathway). Patients will undergo CXR and US at this visit prior to intervention.
- **Day 30:** follow-up by telephone/review of hospital records to determine further pleural procedures, emergency hospital attendances, other treatment for effusion *e.g.*, chemotherapy, surgery, diuretics.
- **Three months:** follow-up by telephone/review of hospital records to determine further pleural procedures, emergency hospital attendances, other treatment for effusion *e.g.*, chemotherapy, surgery, diuretics. We will also collect health related QoL data using the EQ-5D 5L questionnaire by telephone.

Research staff will informally assess capacity at each follow-up visit. If the investigator believes that the participant has lost capacity to consent, no further data will be collected, and they will be withdrawn. The study data and samples will be used/retained if the participant had consented for this during the initial consent process.

### Pleural fluid and blood Sample handling (optional long-term storage)

Pleural fluid and blood samples will be processed after taking and centrifuged according to the study specific procedures. They will then be stored locally in a freezer at -80°C. Samples will be stored and may be used for exploratory analysis such as proteomics analysis and measurement of individual biomarkers.

### Discontinuation/withdrawal of participants from study

Participants may withdraw from the study at any time. The research team may also withdraw a participant if necessary, for example for the following reasons:

- Identification of exclusion criteria *e.g.*, pleural infection
- Inability to perform therapeutic aspiration
- Pregnancy
- Significant protocol deviation
- Withdrawal of consent
- Loss to follow-up

If provided, the reason for withdrawal will be recorded in the CRF. Data and samples collected prior to withdrawal will be included in analysis unless the participant requests that they are not used. If the participant loses capacity and they had not consented for their study data/samples to be retained following loss of capacity to consent, their data/samples will not be used.

### Definition of end of study

The end of study is the date when all sample analysis is complete.

### Distress protocol

We recognise that patients attending the Pleural Clinic may find the experience distressing, due to anxiety over the possible cause of their pleural effusion or having to undergo an invasive procedure. They may become distressed during trial procedures, and it is imperative that the researcher responds to this distress in a sensitive and reassuring manner.

The distress protocol is as follows:

1. A participant indicates that they are experiencing a high level of stress or emotional distress OR exhibits behaviours suggesting the trial procedures are too stressful *e.g.*, uncontrolled crying, shaking.
2. Stop the trial procedures. One of the researchers or clinical team will offer immediate support by reassuring the patient and exploring the reason for their distress.
3. If the participant feels able to carry on, then trial procedures can be continued. It is important that the participant is aware they can withdraw from the trial at any point.
4. If the participant feels unable to carry on, then stop trial procedures and continue to reassure them. Remind them of the support that is available to them during this process *e.g.*, from the specialist nursing team.
5. If the participant requests to withdraw from the study, then their wishes will be respected.

### Statistics and data analysis

***Description of statistical methods***

Reporting will include the number of patients screened for inclusion, the number of participants completing the study and exploratory analysis of any correlations between baseline factors and change in size of effusion on CXR.

***Sample size and analysis plan***

Based on our feasibility data, we estimate we need to recruit 200 patients to have complete data available on 150 patients. The main reason for this is that approximately 20% of patients will turn out not to have MPE and therefore will not be eligible for inclusion in the final analysis. The primary outcome measure is at one week and is part of standard clinical care and therefore we anticipate that loss to follow-up, withdrawal rates and missing data will be minimal based on our feasibility data and previous trial experience. A further validation cohort of 40 patients will be recruited subsequently. Follow-up will be either by review of available medical records, telephone or clinical appointment (if patient is coming up to hospital anyway) at 30 days and three months.

A sample size of 150 participants would allow us to have a shrinkage factor of 0.95, which is above the recommended 0.9, assuming an R ^2^ value of 0.5 and 8 parameters in the model. It would also allow the estimated R ^2^ value to be within recommended limits of 0.05 of the true R ^2^ value. It would allow the estimation of residual standard deviation to be within 13%. Although this is above the recommended 10%, we believe that it is still clinically meaningful. It also allows the estimation of the mean outcome to within +/- 1.5%, which is sufficiently precise to be clinically useful, assuming an overall mean of 14 and an SD of 13. This represents 18.75 individuals per variable, which is within the traditional 10–20 individuals per variable rule-of-thumb. The analysis will be based on traditional regression methods and follow the Transparent reporting of a multivariable prediction model for individual prognosis or diagnosis (TRIPOD) statement ^16^. An outline of the analysis is as follows:

1. Initial screening of potential predictor variables to ensure that there is sufficient variation in them and that they are not missing at an unreasonably high level. For example, if a categorical variable does not have >5% of participants in any category; or if a continuous variable does not cover a broad enough range. After the initial screening, if appropriate data reduction techniques such as principal component analysis will be undertaken to reduce the number of variables to a reasonable amount.
2. Model selection will then be undertaken using variable selection such as backwards elimination. Continuous covariates will be checked for non-linear relationships using splines.
3. Once an appropriate model has been selected, the optimism and internal validation will be estimated using a non-parametric bootstrap approach. If overfitting if found to be an issue, then penalised regression (Lasso or Ridge regression) will be used to reduce the amount of overfitting.
4. Once the final prediction model has been estimated, its properties such as discrimination by the estimation of the R ^2^ and calibration by plotting the observed outcome against the predicted outcome will be explored. If there is a large number of participants excluded due to missing predictor variables, then the sensitivity of the final prediction model to missing data will be assessed using multiple imputation.

***Procedure for accounting for missing, unused, and spurious data***

Rates of missing data will be reported. For the purposes of this exploratory study, if a patient is “lost to follow-up” or dies before the three-month outcome point, it will be assumed (unless hospital records indicate otherwise) that no further pleural procedure has occurred.

***Inclusion in analysis***

All participants with available data will be included in the day seven analysis. For the one month and three-month outcomes, all participants including those lost to follow-up or who die within the time period will be included in analysis.

***Assessment of effusion size on CXR***

Percentage opacification of the hemithorax by effusion will be measured on the post-procedure and follow-up CXR. This is a validated measure of effusion size performed using freely available software ( PaintShop Pro 2022 (RRID:SCR_000338), Corel Corporation and ImageJ version 1.53r (RRID:SCR_003070), National Institutes of Health (RRID:SCR_011417)). Anonymised digital CXR images will be uploaded onto the study database. PaintShop Pro is used to outline the hemithorax and effusion. These shapes are then converted into a pixel count by ImageJ. Percentage opacification is calculated by dividing the effusion pixel count by the hemithorax pixel count. This analysis takes approximately five minutes per image.

### Data management

***Source data***

Source documents are where data are first recorded, and from which participants’ CRF data are obtained. These include, but are not limited to, hospital records (from which medical history and previous and concurrent medication may be summarised into the CRF), clinical and office charts, laboratory and pharmacy records, diaries, microfiches, radiographs, and correspondence.

CRF entries will be considered source data if the CRF is the site of the original recording ( *e.g.*, there is no other written or electronic record of data). All documents will be stored safely in confidential conditions. On all study-specific documents, other than the signed consent, the participant will be referred to by the study participant number/code, not by name.

***Access to data***

Direct access will be granted to authorised representatives from the Sponsor, host institution and the regulatory authorities to permit study-related monitoring, audits and inspections. Data will be made available as an online supplement at the time of publication.

***Data recording and record keeping***

All study data will be entered on to a web-based data management system ( REDCap (RRID:SCR_003445)).

The study database is bespoke and hosted on the University of Oxford server with services provided through Oxford University Medical Sciences Division IT Services. The server and database are protected by a number of measures including anti-virus and anti-spyware applications, firewalls, encryption technology and permissions. The database will be backed up on a daily basis. The Data Manager will maintain a list of personnel with authorization to edit and/or view data. All study data will be archived for five years.

The database and access to computers are password protected. Paper-based identifiable data at each site will be kept in a locked cabinet, in a locked or ID-access controlled area this will be kept for 6–12 months post the end of the study only if the participant consents to being informed of the study results. If participants do not consent to this, identifiable information will be destroyed once data analysis has taken place.

The participants will be identified by a unique trial specific number in any database. The name and any other identifying detail will not be included in any study data electronic file.

### Quality assurance procedures

The study will be conducted in accordance with the current approved protocol, principles of GCP, relevant regulations and standard operating procedures. The Trial Management Committee (consisting of the trial manager and chief investigator) will meet monthly to ensure the trial is running as planned.

Regular monitoring will be performed according to principles of GCP. Data will be evaluated for compliance with the protocol and accuracy in relation to source documents. Following written standard operating procedures, the monitors will verify that the clinical study is conducted, and data are generated, documented and reported in compliance with the protocol, principles of GCP and the applicable regulatory requirements.

### Ethical and regulatory considerations

***Declaration of Helsinki***

The Investigator will ensure that this study is conducted in accordance with the principles of the Declaration of Helsinki.

***Guidelines for GCP***

The Investigator will ensure that this study is conducted in accordance with relevant regulations and principles with GCP.

***Approvals***

The protocol, informed consent form and participant information sheet have been submitted to an appropriate Research Ethics Committee (REC), and written approval was given on 15 ^th^ June 2021 (Ethics Ref: 21/PR/0607; IRAS Project ID: 295614).

The Investigator will submit and, where necessary, obtain approval from the above parties for all substantial amendments to the original approved documents.

***Reporting***

The Chief Investigator shall submit once a year throughout the clinical study, or on request, an Annual Progress Report to the REC, host organisation and Sponsor. In addition, an End of Study notification and final report will be submitted to the REC, host organisation and Sponsor.

***Participant confidentiality***

The study staff will ensure that the participants’ anonymity is maintained. The participants will be identified only by a participant ID number on all study documents and any electronic database, with the exception of the CRF, where participant initials may be added. All documents will be stored securely and only accessible by study staff and authorised personnel. The study will comply with the Data Protection Act, which requires data to be anonymised as soon as it is practical to do so.

***Expenses and benefits***

All visits are part of standard clinical care so no travel expenses will be offered to patients.

### Coronavirus disease 2019 (COVID-19) risk mitigation

In the light of the current COVID-19 pandemic, it is essential that research participants and staff are kept safe and not exposed to any unnecessary risk. The participants eligible for enrolment into REPEAT are a high-risk group, because they may have disseminated cancer (albeit undiagnosed at the time of recruitment). REPEAT has been designed to align with standard clinical care. The participant will have two face-to-face hospital visits, one for initial pleural fluid drainage and the second for results and reassessment. Both are essential visits. All other follow-up visits can be done by telephone. All staff and participants will wear masks, practice social distancing where possible and perform good hand hygiene, all as standard care. Therefore, the participants or staff will not be exposed to any increased risk of COVID-19 infection while participating in REPEAT.

A further risk from COVID-19 is to the recruitment success of the trial. Recruitment will start in the second quarter of 2021, by which time we anticipate coming to the end of the predicted Winter peak of COVID-19 infections. Therapeutic aspiration is an essential, emergency procedure that prevents hospital admission. Therefore, Pleural Clinics have continued to provide this procedure during the COVID-19 pandemic, and we anticipate this will continue. Thus, we do not predict a decrease in pleural procedures due to COVID-19.

Finally, there is the risk of lack of availability of research staff to support recruitment and data collection. However, the recruiting centres all integrate their research and clinical teams, and we anticipate that we will be able to recruit successfully.

### Finance and insurance

***Funding***

This study is funded by a National Institute for Health Research - Research for Patient Benefit grant.

***Insurance***

NHS bodies are legally liable for the negligent acts and omissions of their employees. If subjects are harmed whilst taking part in a clinical study as a result of negligence on the part of a member of the study team this liability cover would apply.

Non-negligent harm is not covered by the NHS indemnity scheme. The Norfolk and Norwich University Hospital NHS Foundation Trust, therefore, cannot agree in advance to pay compensation in these circumstances.

In exceptional circumstances an *ex-gratia* payment may be offered.

### Publication policy

Once completed, this study will be submitted to a conference as an abstract and as a paper to a peer-reviewed journal.

## Study status

As of 24 ^th^ June 2022, five sites are currently recruiting to the study and 62 subjects have been recruited. Five more sites are currently in set up.

## Data Availability

No data are associated with this article. Extended data Zenodo: REPEAT protocol extended data. https://doi.org/10.5281/zenodo.7551597 ^16^ This project contains the following extended data: - Full_CRF_pack.pdf - REPEAT_Consent_form_WithSamples_V2.0_28May2021_NNUH.doc - REPEAT_PIS.docx - REPEAT therapeutic aspiration SOP.docx - REPEAT ultrasound SOP.docx - REPEAT_VAS_diary.pdf Data are available under the terms of the Creative Commons Attribution 4.0 International license (CC-BY 4.0).
