# Supplementary material for: Trial Protocol: Reaccumulation rate of pleural effusions after therapeutic aspiration: An observational cohort study to determine baseline factors associated with rate of pleural fluid reaccumulation following therapeutic aspiration in patients with malignant pleural effusion attending a pleural clinic (REPEAT)
Source: NIHR Open Res. 2023 Jan 23;3:5. [Version 1] doi: 10.3310/nihropenres.13282.1 (PMC10593318; doi:10.3310/nihropenres.13282.1)
Supplement: Peer review history

# Reviewer report: Reviewer response for version 1

**DOI:** 10.3310/nihropenres.14402.r30279

**Published:** 2023-10-09

**Authors:** Neslihan Ozcelik

**Affiliations:** 1 Faculty of Medicine, Chest Disease, Recep Tayyip Erdogan University, Rize, Turkey

Overall, the article proposal is well-written and well-conceived. The research question is clear and important, and the proposed methods are appropriate. The anticipated impact of the study is significant, as it could help patients and clinicians to make better decisions about the treatment of MPE.

- The research question is clear and focused.
- The proposed methods are appropriate and feasible. The sample size of 200 patients is sufficient to develop a reliable prediction model. The proposed clinical score is based on common clinical biomarkers that are easy to collect.
- The anticipated impact of the study is significant. The ability to predict the rate of reaccumulation of MPE could help patients and clinicians to make better treatment decisions and improve the quality of care.

Minor comments:

- It would also be helpful to provide more information about the clinical score that will be developed. For example, what specific clinical biomarkers will be included in the score? How will the score be weighted?
- Finally, it would be helpful to discuss the potential limitations of the study. For example, the study will only include patients with MPE who are undergoing therapeutic aspiration. It is possible that the prediction model will not be generalizable to other patients with MPE.

Is the study design appropriate for the research question?

Yes

Is the rationale for, and objectives of, the study clearly described?

Yes

Are sufficient details of the methods provided to allow replication by others?

Partly

Are the datasets clearly presented in a useable and accessible format?

Not applicable

Reviewer Expertise:

Pulmonology, Lung Cancer, Pleural Disease, Artificial intelligence in medicine

I confirm that I have read this submission and believe that I have an appropriate level of expertise to confirm that it is of an acceptable scientific standard.

# Reviewer report: Reviewer response for version 1

**DOI:** 10.3310/nihropenres.14402.r30273

**Published:** 2023-09-18

**Authors:** Apostolos G Pappas

**Affiliations:** 1 National and Kapodistrian University of Athens, Athens, Greece

The authors will try to evaluate a very important clinical question with a well-designed protocol. More specifically, they will try to develop a predictive score for the risk of rapid re-accumulation of MPE by combining clinical, epidemiological and imaging data with pleural fluid and serum biomarkers. In my opinion, two key components of the protocol are the following:

 a) the inclusion of a validation cohort of 40 patients,

 b) the evaluation of biomarkers more commonly associated with heart or renal failure, which are two modalities that may co-exist with MPE and thus lead to the rapid accumulation of pleural fluid.

**Minor comment:**

 As it is thoroughly explained in the introduction, vaso-active mediators promote mesothelial hyper-permeability, a hallmark of MPE formation. The authors state in the "methods" that pleural fluid and serum samples "will be stored and may be used for exploratory analysis such as proteomics analysis and measurement of individual biomarkers". For this reason it is reasonable to assume that vaso-active mediators will be investigated as candidate biomarkers for the development of the final model. However, this remains unclear from the protocol description.

Is the study design appropriate for the research question?

Yes

Is the rationale for, and objectives of, the study clearly described?

Yes

Are sufficient details of the methods provided to allow replication by others?

Yes

Are the datasets clearly presented in a useable and accessible format?

Not applicable

Reviewer Expertise:

Pleural disease; Mesothelioma

I confirm that I have read this submission and believe that I have an appropriate level of expertise to confirm that it is of an acceptable scientific standard.

# Reviewer report: Reviewer response for version 1

**DOI:** 10.3310/nihropenres.14402.r29817

**Published:** 2023-07-12

**Authors:** Andrea Ban Yu-Lin

**Affiliations:** 1 Universiti Kebangsaan Malaysia Medical Centre, Kuala Lumpur, Malaysia

The study is relevant. As the clinical question is one we frequently ask and try to predict. It is brilliant that the authors are addressing this in their study. I look forward to the results of this trial when it is published.

 No major comments.

 Minor comments.

- I suggest the inclusion criteria be detection of pleural effusion confirmed via ultrasound. My reasons are if it is detected only on CT scan, it is possible that the effusion is minimal.
- Patients will bilateral pleural effusions may presenting for therapeutic tap. In this scenario, the authors should address whether they will do a diagnostic tap on the larger effusion or these patients will be excluded from the study.
- Loculated effusions are possible on first presentation although uncommon. But if they present, would the authors still include them in the study?
- The authors stated that only MPEs will be included into the study and analysed. Criteria for diagnosing MPE  is based presumably on presence of malignany cells on cytology. Would the authors also consider including MPE with obvious clinical diagnosis but no presence of malignant cells?

This study is well described. 

 I am unable to comment on the statistics as this is not my expertise.

Is the study design appropriate for the research question?

Yes

Is the rationale for, and objectives of, the study clearly described?

Yes

Are sufficient details of the methods provided to allow replication by others?

Yes

Are the datasets clearly presented in a useable and accessible format?

Yes

Reviewer Expertise:

Pulmonologist.

I confirm that I have read this submission and believe that I have an appropriate level of expertise to confirm that it is of an acceptable scientific standard.

# Response

**Published:** 2023-07-20

**Authors:** Eleanor Mishra

**Affiliations:** Norfolk and Norwich University Hospitals NHS Foundation Trust, UK

Thank you for your review. I'm pleased to say that we have recently completed recruitment and are now working on completing follow up and cleaning data for this study.

 1. Re. inclusion criteria: patients had to be attending for therapeutic aspiration (large volume drainage) of their effusion to prevent enrolment of patients with minimal effusions.

 2. Patients with bilateral effusions were included in the study. In this instance, clinicians would chose which side to drain (usually the larger) and document this on the CRF.

 3. Patients with loculated effusions are included in the study. This would be visible on the study ultrasound and documented on the CRF.

 4. The criteria for diagnosis of an MPE is the final diagnosis from the clinician, including both malignant cells on cytology and a clinical diagnosis in the absence of cytology/histology. 

 Thanks again for your feedback. I hope to have results of this study at the end of the year.
